# Investigating the Structural and Functional Consequences of Pathogenic SNPs on Human VEGFA Dimer: Insights from Molecular Dynamics Study

**DOI:** 10.1021/acsomega.5c07106

**Published:** 2025-10-28

**Authors:** Rajib Islam, Md. Jahirul Islam, Sadia Jaman, Md. Arafat Hossen, Sayeda Samina Ahmed, Syeda Samira Afrose, Md. Junaid, Md Shahinozzaman, Mohammad A. Halim

**Affiliations:** † Division of Computer-Aided Drug Design, The Red-Green Research Centre, BICCB, 16 Tejkunipara, Tejgaon, Dhaka 1215, Bangladesh; ‡ Genomic and Proteomic Research Division, ABCD Laboratory, Bangladesh, Chattogram 4226, Bangladesh; § Division of Pharmaceutical Quality Research, Office of Pharmaceutical Quality Research, Center for Drug Evaluation and Research, 4137 U.S. Food and Drug Administration, Silver Spring, Maryland 20993, United States; ∥ Department of Chemistry and Biochemistry, 271594 Kennesaw State University, Kennesaw, Georgia 30114, United States

## Abstract

Vascular endothelial growth factor A (VEGFA) is a key regulator of angiogenesis. It forms a homodimer and binds to VEGF receptors to activate signaling pathways important for blood vessel growth and maintenance. In this study, we examined the structural and functional effects of two deleterious mutants, R262Q and C266Y, using integrative computational methods. Molecular dynamics simulations and principal component analysis showed that both mutations disrupted crucial interface interactions, including H-bonds, salt bridges, and disulfide linkages. As a result, the dimer conformation became less stable, which was also supported by binding free energy analysis. In addition, structural superimposition analysis revealed that both mutations changed the receptor-binding shape. This may block normal signaling and affect biological functions. Overall, our findings provide structural insights into how these mutations affect VEGFA dimer and may help guide the development of new therapeutics.

## Graphical abstract

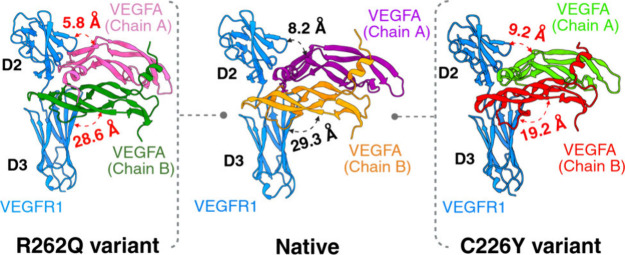

## 1 Introduction

Vascular endothelial growth factor A (VEGFA) gene, also known as VPF (vascular permeability factor) or MVCD1 (microvascular complications of diabetes type 1), belongs to the platelet-derived growth factor (PDGF/VEGF) family and is uniquely positioned on chromosome 6p21.1, having eight exons and seven introns.^1^ This factor has been identified for its role in vascular permeability, with activity shown in tumor cells from rodents.^2^ In earlier studies, multiple research teams found that VEGFA uniquely facilitated the movement of vascular endothelial cells and induced angiogenesis *in vivo*.^3^ Based on these findings, factors associated with this activity were renamed and categorized as part of the VEGF family.^4^

The VEGF family consists of five members that significantly impact the human cardiovascular system. VEGF-A binds to and activates both receptors: VEGFR-1 and VEGFR-2, facilitating angiogenesis, enhancing vascular permeability, stimulating cell migration, and influencing gene expression.^5^ Additionally, Lee et al. demonstrated that an autocrine loop involving VEGF-A and its receptors exists within vascular endothelial cells, playing a crucial role in maintaining endothelial functions.^6^ Uciechowska-Kaczmarzyk et al. reported that the linker is predominantly intrinsically disordered, but upon binding to heparin at the heparin-binding domain (HBD), VEGFA undergoes a conformational reorganization in which the two HBDs fold around heparin to form a hairpin-like sandwich structure. This arrangement not only altered VEGFA’s conformational ensemble but also modulated VEGFA–VEGFR2 recognition.^7^ VEGF-B is crucial for cardiovascular development, embryonic angiogenesis, and the formation of the embryonic myocardium, as well as promoting blood vessel survival.^8^ VEGF-C and VEGF-D primarily facilitate lymphangiogenesis. Additionally, placental growth factor (PIGF) is involved in both angiogenesis and wound healing.^9^

Earlier study reported that disruption of VEGFA gene in mice leads to abnormal formation of embryonic blood vessels. Furthermore, this gene is often upregulated in various tumors, with its expression levels correlating with the stage and progression of the cancer.^10^ Numerous genetic variations have been identified in the VEGFA gene, some of which impact the secretion and regulation of VEGF expression.^11^

Single-nucleotide polymorphisms (SNPs) are the most common genetic variation, responsible for about 90% of human genetic variation. Several loci have been linked to gene phenotypes and an increased risk of tumor development.^12^ SNPs are genetic markers that appear approximately every 200–300 base pairs in the human genome and about 0.5 million SNPs are present in the coding region of the human genome. Substituting amino acids in the coding region of genes can impact the structure, stability, and function of proteins. Nonsynonymous SNPs (nsSNPs) that pose a significant risk of causing mutations or altering protein function are referred to as high-risk nsSNPs.^13^

In recent years, computational algorithms have been widely used to assess the effects of harmful nsSNPs in candidate genes, utilizing data such as sequence conservation across species, structural and functional properties of polypeptides.^14^ Using computational approach, several studies have successfully identified highly functional SNPs from a huge pool of disease-susceptible SNPs of STK11,^15^ BMPR1A,^16^ and KRAS^17^ genes by analyzing their structural and functional impacts.

In this study, we employed several *in silico* tools to screen nsSNPs, aiming to identify the most deleterious nsSNPs in the human VEGFA protein. We also assessed their impact on the structure and function of VEGFA, providing deeper insights of its dynamic properties and potential therapeutic targets or options.

## 2 Materials and Methods

The complete workflow to identify the high risk nsSNPs in human VEGFA gene and their structural/functional consequences using different computational tools and databases are summarized in the Supplementary Figure S1.

### SNP Data Set Collection

The nonsynonymous single nucleotide polymorphism data of the VEGFA gene were collected from NCBI dbSNP database, Ensembl genome browser and the protein sequence was collected from UniProt database (UniProtKB ID: P15692). These nsSNPs were further analyzed by various bioinformatics tools (Supplementary Table S1).

### Prediction of Functional Consequences of nsSNPs

The functional consequences of nsSNPs were predicted by using SIFT (Sorting Intolerant from Tolerant)^18^ and PolyPhen-2 (Polymorphism Phenotyping-2).^19^ Utilizing the PSI-BLAST algorithm, SIFT server predicts the effect of substitution on protein function based on the homology sequence of the protein and alignment of the naturally occurring nsSNPs with paralogous and orthologous protein sequences. In the SIFT algorithm, a score of less than 0.05 is considered as the deleterious or intolerant impact of nsSNPs on protein function, while a score of more than 0.05 is considered as tolerant. The web-based tool PolyPhen-2 predicts the possible structural and functional impact of an amino acid substitution on the of a human protein using the protein sequence and substitution of amino acids. It is a probabilistic classifier for calculating the functional impact of an allele change. It classifies all the nsSNPs in three categories such that probably damaging (score 0.95), possibly damaging (score 0.45–0.94) and benign (score 0.45).

### Prediction of the Phenotypic Effect of nsSNPs

To identify the functional consequences, nsSNPs were subjected to PredicSNP server^20^ which predicts the result from various established predicting tools like MAPP, nsSNPAnalyzer, PANTHER, PhD-SNP, PolyPhen-1, and SNAP. For predicting the functional effects, MAPP predicts the functional effects of amino acid replacement particularizing the physicochemical variation, while SNAP utilizes the neural network-based method. The nsSNPAnalyzer uses Random Forest method for predicting the phenotypic effect of the mutation, whereas PolyPhen-1 applies empirical functions. Furthermore, PANTHER and PhD-SNP predict the effect of coding nsSNPs using evolutionary relationships and SVM (support vector machine) respectively.

The nsSNPs were further subjected to the SNPs&GO^21^ binary classifier server for predicting the association of single amino acid variations (SAVs) to disease. It discriminates the nsSNPs between disease-related and neutral SAVs by considering protein functional annotation. It gives prediction by combining the protein sequence information with functional annotation encoded by Gene Ontology terms. Its accuracy rate is 81% and tested on more than 38,000 SAVs from the SwissVar data set in sequence mode.

### Prediction of Protein Stability

The I Mutant 2.0 calculates a protein stability change upon single-site mutations according to Gibbs-free energy change (DDG) using Neural Network based predictor.^22^ This SVM based server contains the most comprehensive database of experimental data derived from ProTherm^23^ on protein mutations. It is optimized to predict the protein stability change upon mutation from the structure or sequence of that protein. The results predict an increase or decrease of stability of the protein upon mutation along with RI (Reliability Index) which ranges from zero to ten. DDG value greater than zero indicates an increase in protein stability and less than zero indicates a decrease in protein stability.

### Identification of Mutant nsSNPs in Different Domains

The InterPro Web server^24^ was used for the identification of domains and also for the mapping of nsSNPs in different domains in VEGFA protein. By classifying proteins into families, domains and important sites, InterPro provides functional analysis using a predictive model known as signature (Provided by different databases). It is a powerful integrated database and diagnostic tool that combines protein signatures from all the member databases. For the prediction of domains and motifs, protein sequence in FASTA format or protein ID was submitted in the InterPro web server.

### Identification of Functional nsSNPs in Conserved Regions

The evolutionary conservation of amino acids was predicted by the ConSurf server,^25^ which relies on the phylogenetic relations between the homologous sequences. According to the Bayesian method classifier a score of 1–4 indicates variable, 5–6 is intermediate, and 7–9 is conserved. The VEGFA protein structure or FASTA sequence was submitted to ConSurf and it predicted the conserved patterns to find a conservation score and coloring scheme with structural and functional amino acid residues. Highly deleterious nsSNPs located in conserved regions were chosen from this selection for comprehensive evaluation.

### Molecular Modeling of nsSNPs

The crystal structure of the human VEGFA dimer was obtained from the Protein Data Bank (PDB ID: 3QTK), using chains A and D as the native structure for molecular dynamics (MD) simulations.^26^ To generate the dimer conformations of the mutant proteins, we used AlphaFold2. This is a powerful machine learning-based method that combines biological and physical information from multiple sequence alignments and structural databases to predict highly accurate protein structures based on amino acid sequence.^27^

### Molecular Dynamics Simulation

Molecular dynamics (MD) simulations were conducted using GROMACS version 2023.5 employing the CHARMM36, which offers robust parametrization for proteins, peptides, and biologically relevant polymers.^28,29^ Initial protein structures were prepared using UCSF Chimera^30^ and solvated in a cubic simulation box filled with TIP3P water molecules,^31^ maintaining a minimum 1.0 nm distance between the solute and box edges. To neutralize the system, appropriate counterions (Cl^–^ or Na^+^) were introduced, and ionic strength was adjusted where applicable.

The system was initially energy-minimized using the steepest descent algorithm, followed by the conjugate gradient method in case convergence thresholds (≤1000 kJ/mol·nm) were not met. Equilibration was performed in two phases: a 100 ps NVT ensemble simulation at 300 K using the velocity-rescale (V-rescale) thermostat (τ = 0.1 ps),^32^ followed by 200 ps NPT ensemble equilibration at 1 bar using the Parrinello–Rahman barostat (τ = 2.0 ps). During both steps, positional restraints were applied to prevent structural distortions.

The production MD simulation was run for 1 μs, using a 2 fs time step under periodic boundary conditions in all directions. LINCS was used to constrain bonds involving hydrogen atoms, allowing stable time integration with the leapfrog integrator.^33,34^ Particle Mesh Ewald (PME) was employed for long-range electrostatics with a 1.2 nm cutoff, and van der Waals interactions were similarly truncated at 1.2 nm.^35^ Trajectory data were saved every 200 ps for downstream analysis.

### Principal Component Analysis (PCA)

Different multivariate factors were analyzed by applying PCA method to understand the structural variations present among the native and mutant proteins in the collected MD trajectory data. Five multivariate factors such as bond distances, bond angles, dihedral angles, planarity, van der Waals energies, and electrostatic energies were considered for this analysis which can visualize the hidden energy and structural dissimilarity among different proteins.^36,37^ The last 50 ns of the MD trajectory data were taken for PCA calculation which were first preprocessed using centering and scaling. The equation that arranged the multivariate in an X axis and convert that into a product of two new matrices by reduction is indicated as

$$X=T_{k}P_{k}^{T}+E$$

Where, Tk is the matrix of scores that gives the impression of how samples relate to each other, Pk is the matrix of loadings which accommodates information about variables relation to each other, k is the factor numbers in the PCA model and E is the residual matrix. All the calculation was performed using R, RStudio and in-house development codes. Finally, the plots were generated by using factoextra Package.

### MM/PBSA Calculation

The MM/PB­(GB)­SA approach was used to estimate the binding free energy of a protein–protein dimer using gmx_MMPBSAv1.6.4.^38^ This tool integrates GROMACS-formatted topologies and trajectories with AmberTools, enabling flexible and reproducible postprocessing of molecular dynamics data for end-state free energy estimation.

The binding free energy (ΔG_bind) was computed using the thermodynamic cycle:

ΔG_bind=⟨G_complex⟩−⟨G_receptor⟩−⟨G_ligand⟩ 1

Each free energy term Gx was calculated as

Gx=⟨EMM⟩+⟨Gsolvation⟩−T⟨S⟩ 2

Alternatively, the binding free energy can be expressed as

ΔGbind=ΔH−TΔS 3

Here, ΔH refers to the enthalpy of binding and −TΔSto the entropic cost of complex formation. In most comparative studies, the entropy term is neglected, yielding an effective binding energy that remains useful for relative affinity ranking.

The enthalpic contribution was decomposed as

ΔH=ΔEMM+ΔGsolvation 4

The molecular mechanics energy in vacuum (ΔE_MM) was split into bonded and nonbonded terms:

ΔE_MM=ΔE_bonded+ΔE_nonbonded 5

Where bonded terms include:

ΔE_bonded=ΔE_bond+ΔE_angle+ΔE_dihedral

And nonbonded terms comprise:

ΔE_nonbonded=ΔE_ele+ΔE_vdW

The solvation energy (ΔG_solvation) included polar and nonpolar contributions:

$$\begin{array}{c} \Delta G\text{\_}\mathrm{solvation}=\Delta G\text{\_}\mathrm{polar}+\Delta G\text{\_}\mathrm{nonpolar}\\ =\Delta G\text{\_}\mathrm{PB}/\mathrm{GB}+\Delta G\text{\_}\mathrm{nonpolar} \end{array}$$ 6

The nonpolar contribution was estimated using either a surface area–dependent model:

ΔGnonpolar=NPtension×ΔSASA+NPOFFSET 7

Or by decomposing it into cavity and dispersion terms:

$$\begin{array}{c} \Delta G\text{\_}\mathrm{nonpolar}=\Delta G\text{\_}\mathrm{disp}+\Delta G\text{\_}\mathrm{CAVITY}\\ =\Delta G\text{\_}\mathrm{disp}+\left(\mathrm{CAVITYTENSION}\times \Delta \mathrm{SASA}+\mathrm{CAVITYOFFSET}\right) \end{array}$$ 8

All energy terms were computed using single-trajectory analysis, wherein the receptor, ligand, and complex conformations were extracted from the same MD trajectory to ensure maximal structural consistency.

In the above equations, ΔEMM represents the molecular mechanical energy in the gas phase, comprising both bonded (internal) and nonbonded (van der Waals and electrostatic) interactions. The solvation energy varies based on the method used: while 3D-RISM computes both polar and nonpolar components, the PB and GB models calculate only the polar part.^39^ The nonpolar term is typically approximated as proportional to the solvent-accessible surface area (SASA), using empirical parameters (Amber’s inp = 1 model). Alternatively, the inp = 2 approach separates the nonpolar term into cavity and dispersion contributions, correlating SASA with the cavity component and using a surface integration method for the dispersion of energy.^40^

## 3 Results

### SNP Data Set Collection

All the SNPs were retrieved from NCBI (National Center for Biotechnology Information) dbSNP database using stepwise filtration. A total of 4578 SNPs was mapped in human VEGFA gene, out of which 3109 SNPs were found to be intron variants, 471 as synonymous variants, 850 (449 in 3′UTR and 391 in 5′UTR) were found to be UTRs variants, and 312 (301 missense and 11 nonsense) were nsSNPs. According to these data nsSNPs contributed to only 6.58% (missense) and 0.24% (nonsense) of all the SNPs mapped in human VEGFA gene (Supplementary Figure S1). In this study, we concentrated primarily on nsSNPs for in-depth investigation, as these variants lead to changes in the encoded amino acids.

### Identification of Deleterious nsSNPs in VEGFA

Several computational tools were used for identifying deleterious nsSNPs in the VEGFA protein. For the initial filtration of nsSNPs from a large pool of SNPs, SIFT and PolyPhen-2 server were employed together to narrow down and predict highly deleterious nsSNPs, compared to other computational tools. Hicks et al. validated the accuracy of SIFT and PolyPhen-2,^41^ while Min et al. demonstrated the strong performance of these predictors in detecting loss-of-function variants, despite the differing prediction mechanisms of the two programs.^42^ Furthermore, several genes such as WFS1,^43^ SLC30A8,^44^ and HMOX1 ^45^ were also reported using these tools in combination, which strongly suggest that using multiple tools is preferable to relying on a single tool for identification.

Out of 312 nsSNPs, SIFT analyzed 160 nsSNPs as deleterious with TI score ≤ 0.05, while 106 nsSNPs as tolerating with TI score >0.05. The rest of the nsSNPs were not found by the SIFT server. Additionally, all the deleterious and tolerated nsSNPs identified by the SIFT algorithm were submitted to the PolyPhen-2 server. According to Supplementary Table S2, 48 nsSNPs were identified as probably damaging in both the HumDiv and HumVar classifiers with a score of ≥ 0.95. The remaining nsSNPs were classified as possibly damaging or benign. The predicted results from SIFT and PolyPhen-2 were categorized into Class 1–3, and 42 highly deleterious nsSNPs in the Class-1 category were selected.

### Phenotypic Effect Prediction for nsSNPs

The 42 nsSNPs selected from the SIFT and PolyPhen-2 analyses were subjected to further validation using the PredictSNP, MAPP, PhD-SNP, PolyPhen-1, SNAP, nsSNP-Analyzer, PANTHER, and SNPs&GO algorithms. To comprehensively understand the phenotypic effects of these nsSNPs, it is recommended to use at least five or six algorithms to ensure accuracy in the predictions. The PredictSNP server incorporates six additional tools, as described in the methods section. Out of the 42 nsSNPs, 8 were found to be deleterious through these seven algorithms (Supplementary Table S3). Ultimately, the combined predictions from PredictSNP, MAPP, PhD-SNP, PolyPhen-1, SNAP, nsSNP-Analyzer, PANTHER, and SNPs&GO algorithms identified five nsSNPs (V239M, P66L, V78M, R82Q, C86Y), as shown in the Supplementary Table S3. These mutants have the Ensembl ID: ENSP00000230480. The positions of these mutants were converted (V239M, P246L, V258M, R262Q, and C266Y) according to the VEGFA_HUMAN sequence (UniProtKB ID: P15692) as shown in the alignment link: P15692:5t89_V.

### Analysis of Protein Stability Due to Mutation

The recent studies reported that the reduction of protein stability can increase misfolding, degradation and aggregation of proteins.^46^ The I Mutant Suite server predicted the protein stability changes upon single point protein mutation from the protein structure and protein sequence. All the five selected nsSNPs (C266Y, P246L, R262Q, V258M, and V239M) were found to decrease the stability of VEGFA protein in I Mutant Suite (Supplementary Table S4) and thus, these nsSNPs might create maximum damage by affecting the protein stability.

### Domain Identification in VEGFA

For identifying the domain region and the location of nsSNPs, the InterPro server was utilized which provides a functional analysis by classifying proteins into families. This server provided 3 domain regions for VEGFA protein: such as the N-terminal domain (1–38), platelet-derived growth factor (PDGF)/VEGF domain (39–135) and C-terminal domain (136–232). Moreover, it was revealed that the selected five nsSNPs (C266Y, P246L, R262Q, V258M, and V239M) are located in the PDGF/VEGF domain (Supplementary Figure S3).

### Evolutionary Conservation Analysis

The intensity of evolutionary conservation at residues in VEGFA protein was identified by employing the ConSurf web server. The ConSurf recognizes structural and functional amino acids and identifies their evolutionary conservation profile based on their location either on the protein surface or inside its core by using the Bayesian classifier method. ConSurf revealed that C266Y, P246L, R262Q, V258M, and V239 M residues are highly conserved with a conservation score of 9 each. It also predicted that V239M, V258M, C266Y residues are the structural and buried residues, whereas P246L and R262Q are the functional and highly exposed residues, as shown in the Supplementary Figure S4. From these mutants, we considered only two mutants, R262Q and C266Y, for further analysis, as these are likely to cause structural disruptions, including the breaking of disulfide bridges and imbalance of electrostatic interactions.

### Molecular Dynamics Simulation of Native and Mutant VEGFA Dimer

To assess the impact of pathogenic mutants in the deviation and stability of PDVG/VEGF domain of VEGFA, we performed 1 μs molecular dynamics simulations for both native and mutants (R262Q and C266Y). As shown in Figure 1a, both mutants exhibited significant deviations from the native protein. RMSD analysis revealed that native VEGFA dimer reached equilibrium states after 120 ns of simulation and maintained until the end of simulation with an average RMSD of ∼ 0.26 nm. However, the R262Q mutant represented an unusual deviation with higher fluctuation (∼0.4 nm) when compared to native. On the other hand, the C266Y mutant exhibited the lowest RMSD (∼0.2 nm) for a prolonged duration of 839 ns, suggesting enhanced rigidity relative to the native structure. However, this stability was slightly disrupted beyond 839 ns, with an increase in deviation reaching ∼ 0.29 nm. These findings highlight distinct dynamic signatures for each variant, suggesting that C266Y might adopt a transiently overstabilized conformation, while R262Q displayed a more flexible and potentially destabilized profile. The corresponding probability distribution of RMSD showed that R262Q had an extended and right-shift distribution compared to native and C266Y, indicating huge conformational mobility that leads to reduced structural stability.

**Figure 1:**
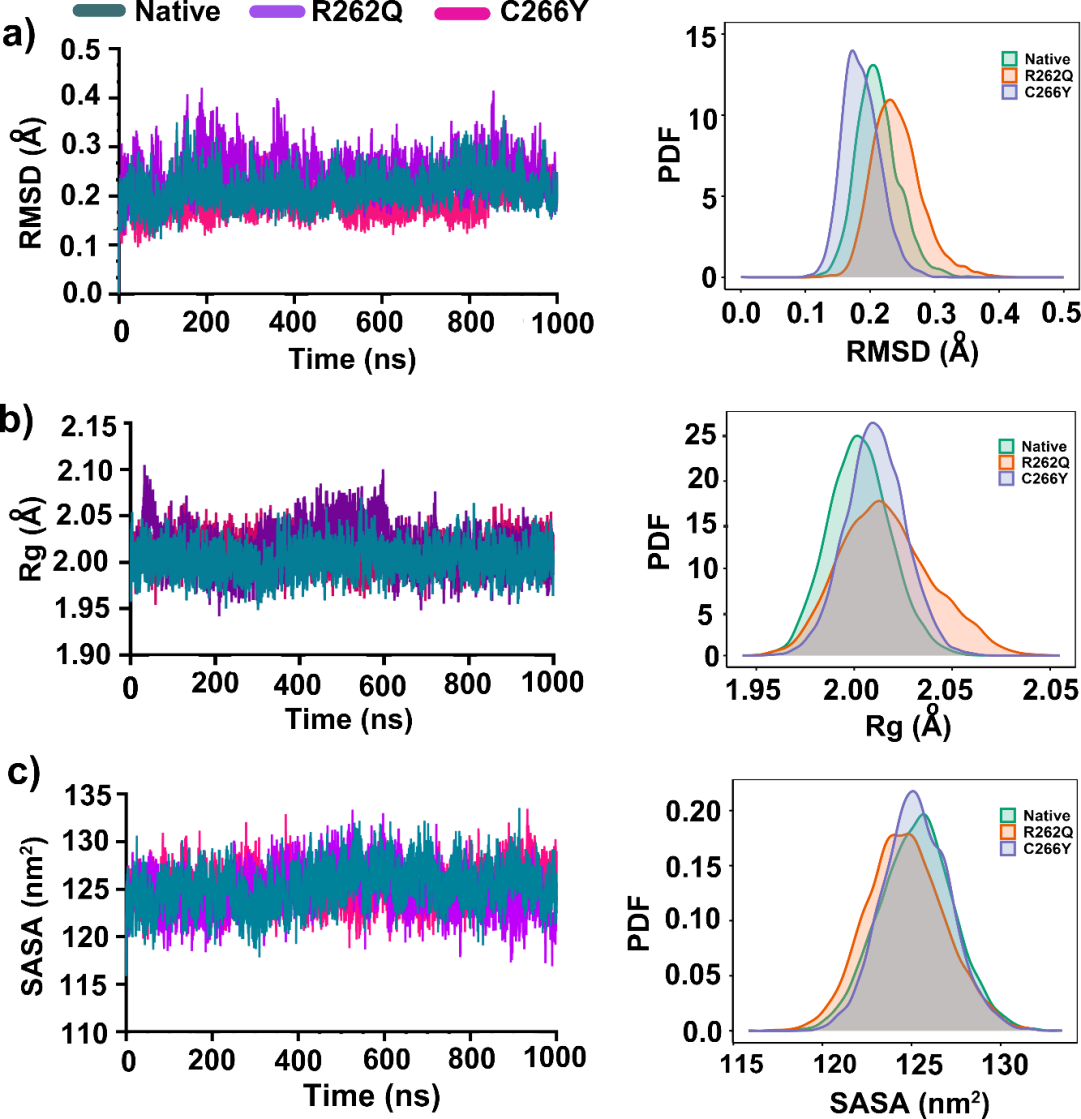
Conformational stability of native, R262Q, and C266Y VEGFA dimers over the course of 1 μs MD simulations. (a) Cα RMSD profiles, (b) Rg profiles, and (c) SASA profiles.

The gyration ratio (Rg) was calculated to assess the compactness of the protein structure, with lower Rg values indicating greater compactness. As illustrated in Figure 1b, the R262Q mutant displayed notable deviations from native, with an increased Rg of ∼ 2.02 nm, suggesting a more expanded and less compact conformation. The highest peak in Rg, reaching 2.08 nm, was observed between 453.1 and 604.8 ns. Although this mutant was stabilized to 1.99 nm after 619.4 ns, its higher overall fluctuation profile implies conformational flexibility that may affect its functional dynamics or interaction potential. In contrast, both native and C266Y exhibited well-converged Rg trajectories throughout the simulation, each stabilizing around 2.01 nm with minimal deviation. The unimodal Rg density distributions for these two further support global compactness and structural consistency of the C266Y variant with the native protein. Notably, the similarity between C266Y and the native form suggests that this mutation does not significantly disrupt the tertiary structural integrity of VEGFA, at least in terms of its overall folding architecture.

The solvent-accessible surface area (SASA) was calculated and depicted in Figure 1c. The SASA data is strongly supported by the Rg findings. All dimer conformations showed relatively stable SASA values during the simulation time, with averages ranging between 120–128 nm^2^. However, the R262Q mutant frequently represented increased SASA values (∼128.3 nm^2^) and higher fluctuations, as confirmed by its expanded and slightly right-shifted PDF distribution (Figure, right panel). This change signifies an expansion of the solvent-exposed surface, likely due to local structural loosening or partial disruption at the dimer interface.

To investigate the local flexibility of native and mutant dimers, we calculated the Cα-RMSF values from the 1 μs simulation trajectory data for A- and B-chain. As shown in Figure 2, there was a distinct flexibility pattern observed between native and mutant dimers. The result clearly revealed that the R262Q mutant exhibited the highest RMSF values across both chains when compared to the native and C266Y structures. This indicates that the R262Q mutation causes significant changes in the residual flexibility of the protein. In the native structure, the most residual flexibility was observed in two loop regions- α2β2 and β4β5, suggesting that these regions are intrinsically flexible (A-Chain). In contrast, the most prominent RMSF fluctuations were seen for R262Q and C266Y in several regions (residues 240–253, 266–272, and 290–293), which lie in the α2-helices, β2-strand, loops like β3−β4 and β4−β5. These results suggest that these structural elements are more flexible in the mutant dimers, especially in R262Q, which may contribute to the overall destabilization of the VEGFA dimer.

**Figure 2:**
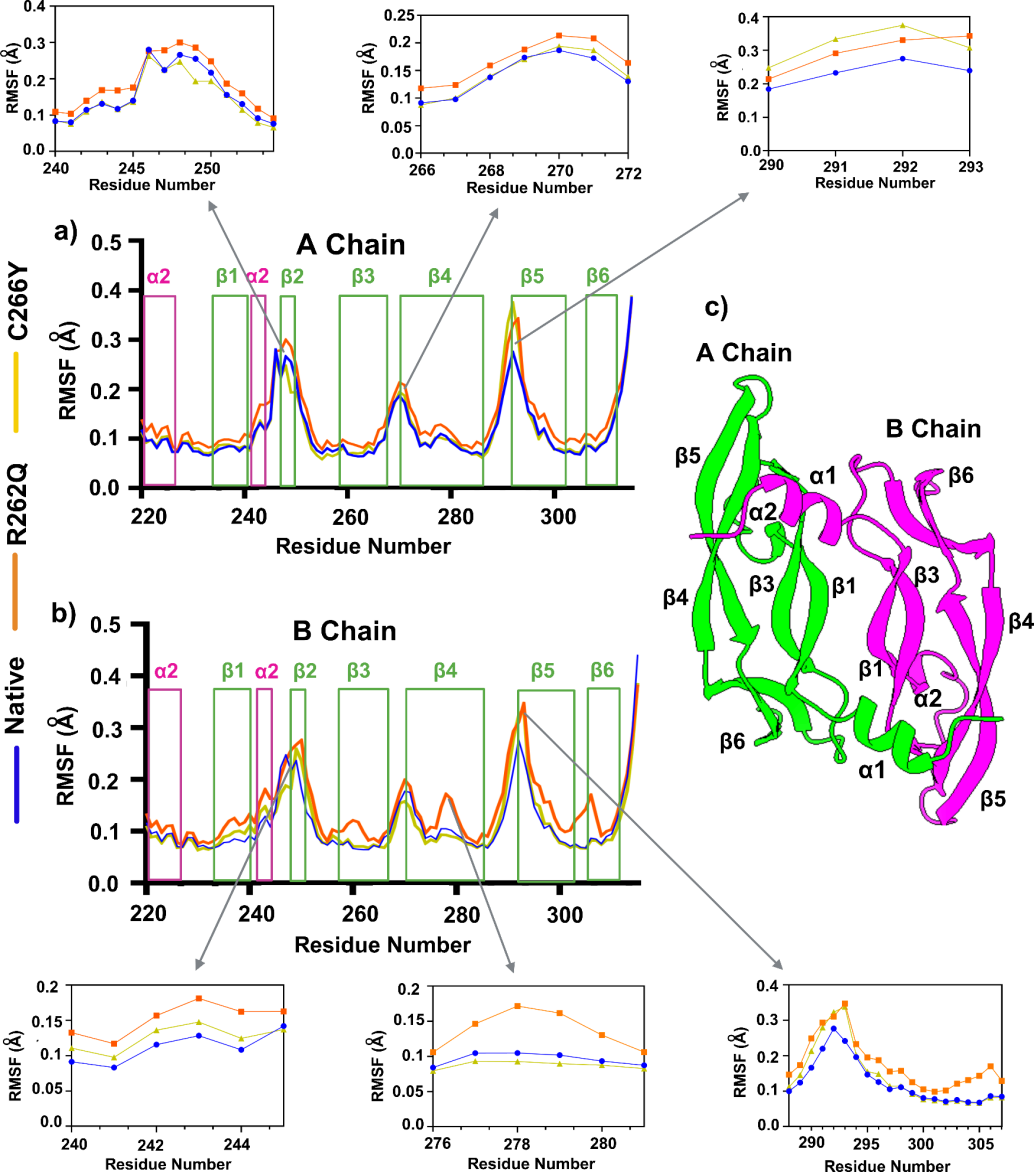
a, b) Residue-wise flexibility analysis of the VEGFA dimer for native, R262Q, and C266Y systems. Secondary structure elements (α-helices and β-strands) are highlighted to indicate fluctuation regions. Zoomed-in plots of selected regions showing higher deviation, including α2−β2, β3−β4, and β4−β5 loops, where mutants exhibit increased flexibility compared to the native structure. c) Cartoon representation of the VEGFA dimer structure, with chain A (green) and chain B (magenta) showing secondary structure elements.

In the B-chain of VEGFA dimer, there was a significant difference fluctuation pattern observed between native and mutants. Notably, the R262Q mutant showed increased fluctuation in several motifs when compared to native and C266Y, suggesting that this mutation likely disrupts local residual flexibility. The most key regions like residues 240–253 (within the α2 helix), 266–272 (partial part of the β3−β4 loop), 276–282 (within the β4 strand), and 288–307 (within the β5 and β6 strand) represented the highest deviations relative to the native structure. This unusual flexibility within the rigid β-strand can lead to loss of structural integrity, which may hinder the β-sheet scaffold, breaking the H-bond network that is crucial for dimer stabilization. This consequence is consistent with earlier reports that higher fluctuations in α-helices and β-strand can impact on the secondary structure stability, particularly when induced by side-chain alterations that disrupt backbone interactions.^47^

### Mutational Effects on VEGFA Dimer Interface Interaction

To investigate how pathogenic mutations impact VEGFA dimer conformation, we calculated interchain H-bond and salt bridge interactions of native and mutants as shown in Figure 3 and Supplementary Table S5. In the native dimer (Figure 3a), the interface was stabilized by forming ten H-bonds and a single functionally significant salt bridge interaction between A:E236 and B:R229 (3.13 Å). Most of the residues like E236, S256, T283, and Q285 at the interface belong to β4−β5 and β6 regions and engage in polar and electrostatic contacts, which contribute to the compact and stable interface of the native dimer. In the case of the R262Q mutant, notable changes were observed at the interface region due to the substitution of charged residue R262Q with polar glutamine, as represented in Figure 3b. Particularly, multiple salt bridges were formed by A:E236 (OE1 and OE2 atoms) with B:R229 (NH1 and NH2 atoms)-resulting in four electrostatic salt bridges forming, while a single one was present in the native conformation. In addition, 14 hydrogen bond interactions were detected at the dimer interface. Among them, key interface residues such as A:V221, A:S256, and A:C266 formed new hydrogen bonds with B:S256, B:C257, and B:T283, respectively, in the R262Q mutant. However, these interactions were absent in native structure. Furthermore, the R262Q mutant formed several new interactions, such as A:S230 – B:C257 and several thiol-mediated polar contacts between cysteine residues that were not present in the native structure. The other mutant C266Y showed a reduced number of hydrogen bond interactions compared to the native structure, forming only seven interfacial H-bond contacts. (Figure 3c). Despite the absence of a key salt bridge interaction (A:E236–B:R229) in the C266Y mutant, a strong hydrogen bonding network was observed between A:K254–B:N268 (1.76 Å) and A:S230–B:C257 (2.57 Å). Moreover, bulky size tyrosine at position 266 formed H-bond interaction with K254, which was not present at position 266 in the native configuration. Overall, the C266Y mutation reduces interaction density and the elimination of key H-bond and salt bridge interactions that could weaken interfacial network.

**Figure 3:**
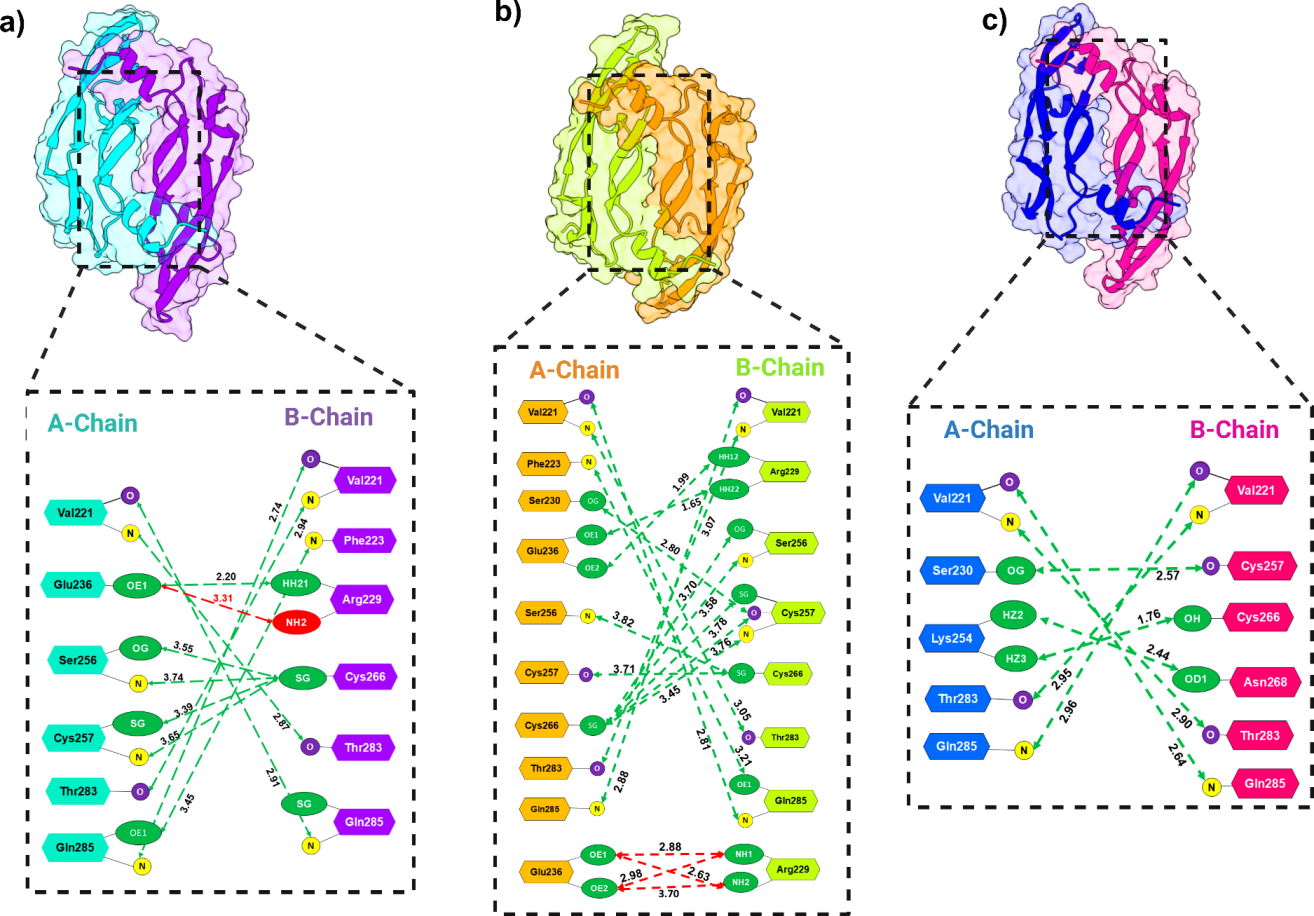
Comparative analysis of dimer interface interactions in native and mutant VEGFA dimers. Interface residues and interaction distances (in Å) are highlighted, with dashed green lines indicating hydrogen bonds and red lines denoting salt bridges (bottom). (a) Native VEGFA dimer showing key hydrogen bonds and one salt bridge (E236–R229) stabilizing the A- and B-chain interface. (b) R262Q mutant dimer exhibits increased hydrogen bonding and formation of multiple salt bridges, altering the interface geometry and potentially overcompensating for local destabilization. (c) C266Y mutant lacks the native disulfide linkage and salt bridge, forming fewer hydrogen bonds and relying on alternative polar contacts, indicating a weakened and structurally altered interface.

### Principal Component Analysis on MD Simulation Data

PCA analysis was conducted based on the structural and energy data from the last 50 ns of 1 μs MD simulation for native and mutants (R262Q and C266Y). For this purpose, we considered bond, angle, dihedral angle, column, and van der Waals as variables. As shown in Figure 4, the first two PCs together explained 54.4% of the total variation in the data, with PC1 contributing 34.9% and PC2 contributing 20.5% of the variance. In the score plot (left panel), the clustering pattern of C266Y deviates significantly to rightward from the native structure, forming a distinct and dense cluster region. This clustering shifting was usual because bond, dihedral, and VdW were the most influential variables along the positive axis of PC1 and PC2 (right panel). However, the R262Q showed partial overlap with the native type, while a major leftward shift was observed along the negative axis of PC1 and PC2. This movement was primarily influenced by Coulomb variable (right panel).

**Figure 4:**
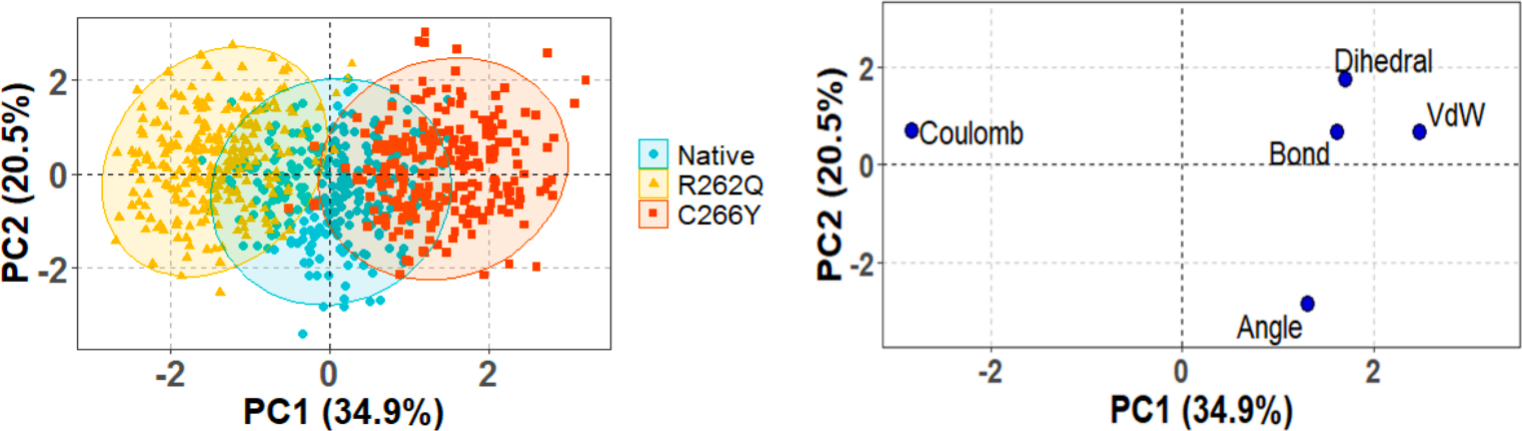
Scores plot presented five data clusters in different colors, where each dot represented one time point. Left panel; the clustering is attributable as Native (cyan), R262Q (light yellow), C266Y (red). Right panel; Loadings plot from Principal Components Analysis of the energy and structural data.

### Binding Free Energy Calculation

To understand the energetic consequences of mutation on VEGFA dimer, we analyzed total binding free energy (ΔG) using MM/PBSA, considering the last 100 ns of molecular dynamics simulation data, as shown in Figure 5. In the native dimer, we observed that the lowest binding free energy, with a ΔG binding value ranging between −98 and −105 kcal/mol. This finding indicates a stable interface maintained in the native dimer, which was consistent with strong H-bonding networks and conserved salt interactions previously identified. In contrast, a higher and abnormal fluctuating binding energy profile was found in R262Q mutant with a total ΔG value ranging from −82 to −93 kcal/mol, indicating a reduction in binding affinity within the dimer chain. Despite the number of H-bond and salt bridge interactions increased, the energy profile suggests that these interactions may be less favorable in nature. However, the C266Y mutant exhibited a moderate binding energy profile, with ΔG values fluctuating between – 94 and – 98 kcal/mol, indicating a mild effect on binding affinity while maintaining overall structural integrity.

**Figure 5:**
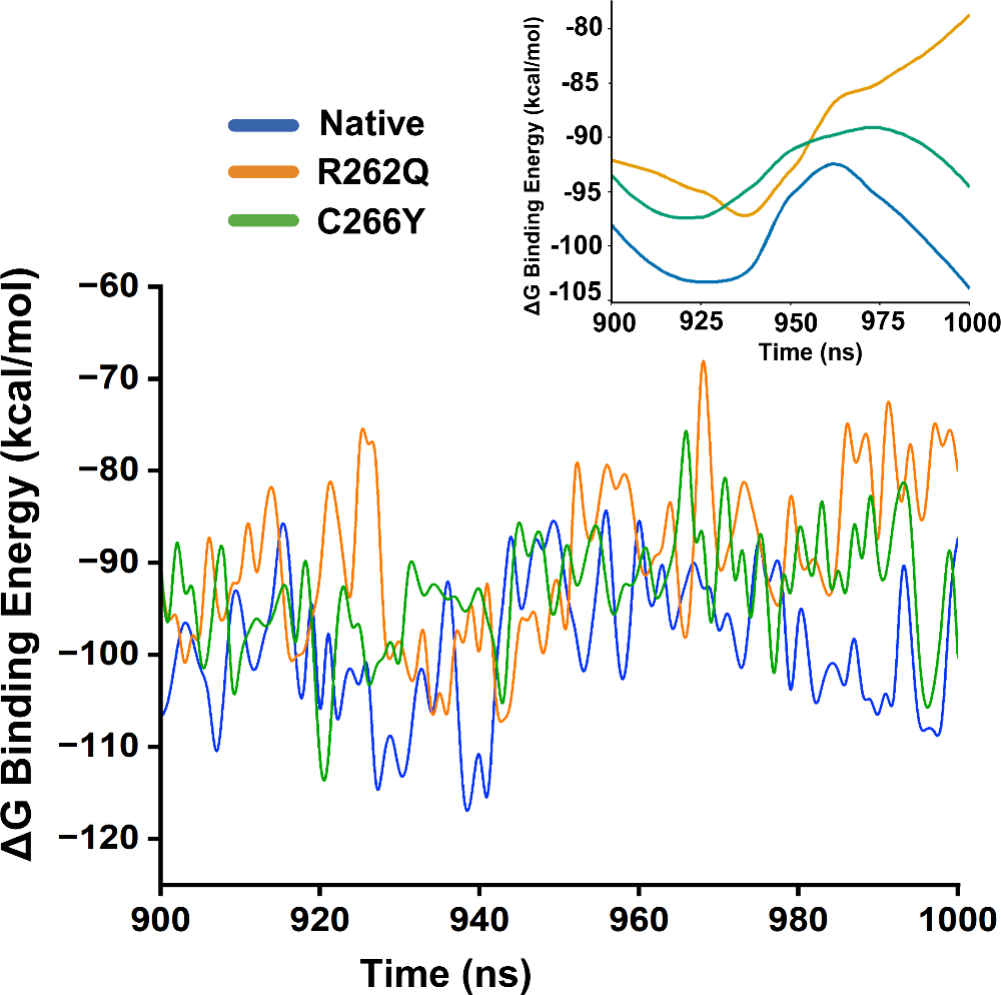
MM-PBSA binding energy analysis calculated from the trajectory of last 100 ns molecular dynamics simulation.

## 4 Discussion

Our study integrated sequence-based and structure-based methods to identify whether specific missense mutants in the PDGF/VEGF domain of VEGFA protein have pathological significance, potentially contributing to rheumatoid arthritis, cancer, and congenital heart disease.^48,49^ The two most significant mutants (R262Q and C266Y) were selected for further analysis according to the combination of different computational algorithms. Prior studies have shown that the PDGF/VEGF domain of VEGFA homodimer binds to VEGF receptors and activates the calcium signaling pathway, calmodulin pathway, and PI3K-AKT pathway that drive angiogenesis and cell growth. Mutation in this domain can change the VEGFR1-binding affinity and disrupt downstream signaling pathways.^50,51^ Notably, the R262Q and C266Y mutants are located in the PDGF/VEGF domain. These residues are highly conserved, surface-exposed, and play an important role in the structure and function of VEGFA. Mutations in the buried residues of a protein can impact its structural integrity, whereas polymorphisms in the exposed residues may alter the protein’s function.^52^ In the R262Q mutation, the native residue arginine is large, positively charged, and hydrophilic, while it is replaced by glutamine, which is smaller, neutral, and polar. The loss of a positively charged arginine at this position may disrupt the local electrostatic interaction and H-bonding contacts, particularly at the dimer interface. This hypothesis is strongly supported by interchain H-bond and salt-bridge analysis, which showed an increase in both types of interactions compared to the native structure. Huang et al. reported that an increased number of H-bonds and salt bridge interactions can lead to a reorganization of the protein’s secondary and tertiary structures, bringing certain amino acid residues closer together and facilitating the formation of additional hydrogen bonds. These new interactions can stabilize the protein in a more rigid conformation.^53^ In addition, RMSF, PCA, and binding free energy analyses collectively suggested that this mutation disrupts residual flexibility and destabilizes the VEGFA dimer. Consequently, it exerts a significant impact on the interface of the VEGFA-VEGFR1 complex. To further support our hypothesis, we superimposed the R262Q mutant onto the native VEGFA dimer (Figure 6). Markovic-Mueller et al. reported that, in the native VEGFR-1/VEGF-A complex, the D2 domain of VEGFR1 primarily mediates hydrophobic interactions with VEGFA residues which are located in the N-terminal α1 helix of protomer A (F223, M224, Y227, Q228, and Y231) and strands β2 (I251, K253), β4 (Q285, M287, I289), and β5 (Q295, I297) of protomer B. These contacts are essential for binding high-affinity and the complex’s proper orientation for downstream signal transduction.^54^ However, R262Q mutant showed a noticeable spatial deviation in both chains from the D2 and D3 domains of VEGFR1 when compared to the native complex, indicating the disruption of critical hydrophobic anchoring (Figure 6e). In the case of C266Y mutant, cysteine belongs to a nonpolar amino acid containing a thiol side chain, whereas tyrosine belongs to a polar aromatic amino acid. Moreover, the tyrosine residue is bulkier and less hydrophobic than the cystine residue, which may not fit at the 266 position. Consequently, this mutant is likely to disrupt hydrophobic contacts in the core of the VEGFA dimer. Notably, in the native structure, A: C266 is involved in forming a disulfide bond with B: C257 at the dimer interface.^26^ Substitution of cystine with tyrosine at this position, the disulfide linkage might be lost, and the VEGFA dimer conformation may be destabilized. A clear insight of stability loss was noticed in the RMSD, Rg, SASA, and RMSF plots, which were also supported by less number of interface H-bond interactions for the C266Y dimer when compared to the native VEGFA dimer. Decreasing H-bond interactions within the interchain of C266Y dimer might assist in losing its rigidity and make it more flexible. Furthermore, there is a conserved salt bridge interaction A: E236-B: R229 located near the N-terminal α1 helix of the native VEGFA dimer interface. This electrostatic interaction is very crucial for maintaining the geometry of the homodimer.^54^ However, C266Y mutant totally disrupted this salt bridge interaction within the dimer interface, which can lead to destabilization of the α1 helix. As a result, misalignment occurs in the residues of the α1 helix and weakening the receptor-binding affinity. Our observation is strongly consistent with the findings of superimposition analysis. As shown in Figure 6f, a noticeable chain deviation has been observed in C266Y mutant, decreasing the distance between C266Y chain B and VEGFR1 D2­(from ∼ 29 Å in native to ∼ 19 Å in mutant), suggesting asymmetric receptor engagement would have occurred. Overall, our study suggests that both mutants significantly disrupt dimer stability by changing interface interactions, which may have a reduced impact on the VEGFA-VEGFR1 binding affinity. As a result, VEGFA’s downstream signaling activation might be lost, leading to impaired angiogenesis, increased endothelial cell death, and imbalanced vascular function.^55^ Multiple studies have previously reported that mutations in the VEGFA dimer protein are associated with several diseases, like autoimmune disease,^56^ recurrent spontaneous miscarriage,^57^ necrotizing enterocolitis,^58^ microvascular complications of diabetes,^59^ metabolic syndrome,^60^ polycystic ovary,^61^ and cancers.^62^

**Figure 6:**
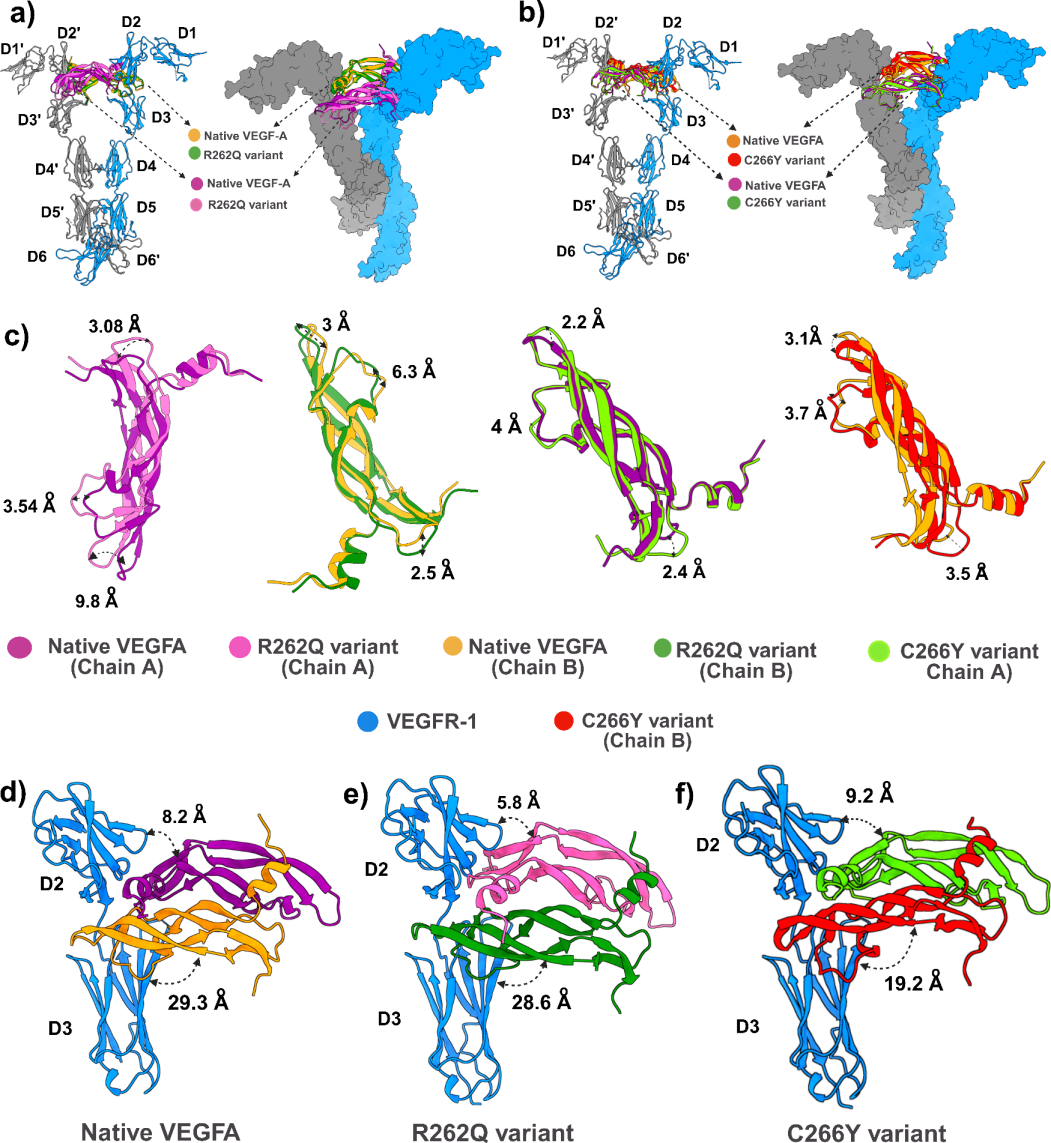
a, b) Structural superimposition of native VEGFA and its R262Q and C266Y mutants, each in complex with the VEGFR-1 extracellular domain. c) Close view of the superimposed VEGF-A structure with the R262Q and C266Y variants reveals transient positional shifts ranging from 2 to 10 Å. d–f) Mutation-induced conformational shift in chain positions compared to the native structure. In the native VEGFR-1-VEGFA complex, the distance between the A chain of VEGF-A and domain D2 of VEGFR-1 is ∼ 8 Å, which is moderately reduced to ∼ 6 Å in R262Q and slightly increased to ∼ 9 Å in C266Y. For chain B, the distance is ∼ 29.3 Å in the native, ∼ 28.6 Å in R262Q, and ∼ 19.2 Å in C266Y, indicating altered binding geometry.

## 5 Conclusion

In this study, we applied a computational approach to investigate the structural and functional impacts of two pathogenic mutants, R262Q and C266Y, within the PDGF/VEGF domain of the VEGFA dimer. Our microsecond molecular dynamics (MD) simulations have revealed that both mutants decrease the dimer’s stability by altering crucial interchain interactions, including H-bonding, salt bridges, and disulfide linkage. Moreover, the results of binding free energy showed a remarkable decrease in binding affinity for both mutants when compared to the native structure, indicating the loss of dimer stability and potentially impacting receptor-binding affinity. Our hypothesis is strongly supported by superimposition and interaction analyses, suggesting that these dimer conformation disruptions may have a negative impact on VEGFA-VEGFR1 complex and downstream signaling. Overall, our findings provide molecular-level evidence that R262Q and C266Y mutants may have pathological impacts on VEGFA’s function, potentially associated with impaired angiogenesis and vascular-related diseases.

## Supplementary Material



## Data Availability

The 3D structures of native VEGFA, R262Q, and C266Y mutant variants, and their complexes with VEGFR1 D2 and D3 (10.5281/zenodo.17215888)

## Notes

Web servers used for nsSNP filtration (Table S1); list of SNPs predicted by SIFT and Polyphen2 (Table S2); list of SNPs predicted by PredictSNP, MAPP, PhD-SNP, PolyPhen-1, SNAP, nsSNP-Analyzer, PANTHER and SNPs&GO (Table S3); protein stability changes due to mutations predicted by I Mutant Suite (Table S4); dimer interface interactions (Table S5) (PDF)

## ⊥

Department of Chemistry, Clemson University, Clemson, South Carolina 29634, USA

## #

Department of Chemistry and Biochemistry, Florida Atlantic University, Boca Raton, Florida 33431, USA
