# Correction to “Investigating the Structural and Functional Consequences of Pathogenic SNPs on Human VEGFA Dimer: Insights from Molecular Dynamics Study”

**DOI:** 10.1021/acsomega.6c02597

**Published:** 2026-04-07

**Authors:** Rajib Islam, Md. Jahirul Islam, Sadia Jaman, Md. Arafat Hossen, Sayeda Samina Ahmed, Syeda Samira Afrose, Md. Junaid, Md Shahinozzaman, Mohammad A. Halim

**Affiliations:** † Division of Computer-Aided Drug Design, The Red-Green Research Centre, BICCB, Tejgaon, Dhaka 1215, Bangladesh; ‡ Genomic and Proteomic Research Division, ABCD Laboratory, Bangladesh, Chattogram 4226, Bangladesh; § PAK Research Center, University of the Ryukyus, Okinawa 903-0213, Japan; ∥ Department of Chemistry and Biochemistry, 271594 Kennesaw State University, Kennesaw, Georgia 30114, United States

In the original publication, the present address for Md. Shahinozzaman was presented as the affiliation. The Author Information of this Correction includes the author’s correct affiliation and present address.

## a

Department of Chemistry, Clemson University, Clemson, South Carolina 29634, USA

## b

Department of Chemistry and Biochemistry, Florida Atlantic University, Boca Raton, Florida 33431, USA

## c

Division of Pharmaceutical Quality Research, Office of Pharmaceutical Quality Research, Center for Drug Evaluation and Research, U.S. Food and Drug Administration, Silver Spring, Maryland 20993, United States

