# Supplementary material for: Investigating the Structural and Functional Consequences of Pathogenic SNPs on Human VEGFA Dimer: Insights from Molecular Dynamics Study
Source: ACS Omega. 2025 Oct 28;10(44):52872–83. doi: 10.1021/acsomega.5c07106 (PMC12613135; doi:10.1021/acsomega.5c07106)
Supplement: Supplementary file 1 [file ao5c07106_si_001.pdf]

Investigating the Structural and Functional Consequences of Pathogenic SNPs on Human VEGFA Dimer: Insights from Molecular Dynamics Study

Rajib Islam[1,$], Md. Jahirul Islam[1], Sadia Jaman[1], Md. Arafat Hossen[1], Sayeda Samina Ahmed[1,¥] , Syeda Samira Afrose[2], Md. Junaid[2], Md Shahinozzaman[3], and Mohammad A. Halim[4*]

[1]Division of Computer-Aided Drug Design, The Red-Green Research Centre, BICCB, 16 Tejkunipara, Tejgaon, Dhaka, 1215, Bangladesh
[2]Genomic and Proteomic Research Division, ABCD Laboratory, Bangladesh, Chattogram, 4226, Bangladesh
[3]Division of Pharmaceutical Quality Research, Office of Pharmaceutical Quality Research, Center for Drug Evaluation and Research, U.S. Food and Drug Administration, Silver Spring, MD 20993, USA
[4]Department of Chemistry and Biochemistry, Kennesaw State University, Kennesaw, Georgia, 30114, USA

Current Address:
[$]Department of Chemistry, Clemson University, Clemson, South Carolina 29634, USA
[¥]Department of Chemistry and Biochemistry, Florida Atlantic University, Boca Raton, Florida 33431, USA

*** Corresponding Author**

Mohammad A. Halim, E-mail: mhalim1@kennesaw.edu

CONTENTS

Table S1. Summary of web servers used for nSNPs filtration

Table S2. List of SNPs predicted by SIFT and Polyphen2

Table S3. List of SNPs predicted by PredictSNP, MAPP, PhD-SNP, PolyPhen-1, SNAP, nsSNP-Analyzer, PANTHER and SNPs&GO

Table S4. Protein stability changes due to mutations predicted by I-Mutant Suite

Table S5. Dimer Interface H-bond and Salt bridge Interactions

Table S1: Summary of web servers used in filtration process of nsSNPS.

Web Server / Tool	Function / Description	URL
**SIFT**	Predicts whether an amino acid substitution affects protein function based on sequence homology.	https://sift.bii.a-star.edu.sg/
**PolyPhen-2**	Predicts the impact of amino acid substitutions on protein structure and function using multiple features.	http://genetics.bwh.harvard.edu/pph2/
**PredictSNP**	Consensus classifier that integrates multiple algorithms to predict the functional impact of variants.	https://loschmidt.chemi.muni.cz/predictsnp/
**MAPP**	Evaluates the physicochemical variation in protein sequences to assess the impact of mutations.	https://mendel.stanford.edu/SidowLab/downloads/MAPP/index.html
**PhD-SNP**	Uses machine learning to classify nsSNPs as disease-related or neutral based on sequence features.	http://snps.biofold.org/phd-snp/phd-snp.html
**SNAP**	Predicts the effect of non-synonymous polymorphisms on protein function using neural networks.	https://rostlab.org/services/snap/
**nsSNP-Analyzer**	Predicts disease-associated mutations by combining sequence and structural features.	http://snpanalyzer.uthsc.edu/
**PANTHER**	Assesses functional impact of mutations based on evolutionary conservation of protein families.	http://www.pantherdb.org/tools/csnpScoreForm.jsp
**SNPs&GO**	Combines protein sequence, function, and evolutionary information to predict disease-related mutations.	https://snps.biofold.org/snps-and-go/
**ConSurf**	Analyzes evolutionary conservation of amino acid positions in a protein sequence.	https://consurf.tau.ac.il/

Table S2: List of SNPs predicted by SIFT and Polyphen2. (D = Damaging, T = Tolerated, Prob. D = Probably Damaging, Poss. D = Possibly Damaging, B = Benign)

Database			SIFT			PolyPhen-2					
**dbSNP**	**Protein ID**	**Mutation**	**Prediction**	**Score**	**Class**	**HumDiv**	**Score**	**Rank**	**HumVar**	**Score**	**Class**
rs45533131	ENSP00000361125	R325G	D	0.05	1	B	0.093	3	B	0.051	3
rs62401172	ENSP00000230480	G116V	T	0.26	3	B	0.151	3	B	0.097	3
rs76869573	ENSP00000230480	P104T	D	0	1	B	0.157	3	B	0.103	3
rs114262569	ENSP00000230480	R80W	D	0	1	Prob. D	0.999	1	Prob. D	0.985	1
rs138551969	ENSP00000361125	K327R	T	0.9	3	Poss. D	0.475	2	B	0.113	3
rs138919899	ENSP00000230480	N8S	T	1	3	B	0	3	B	0	3
rs140461341	ENSP00000230480	R154H	D	0.02	1	Prob. D	1	1	Prob. D	0.985	1
rs141138308	ENSP00000361125	R339W	D	0.01	1	Prob. D	1	1	Prob. D	0.963	1
rs150806902	ENSP00000361125	R339Q	T	0.49	3	Poss. D	0.835	2	B	0.093	3
rs199971699	ENSP00000317598	E179K	D	0	1	Poss. D	0.593	2	B	0.057	3
rs201261787	ENSP00000230480	G6E	T	1	3	B	0	3	B	0.003	3
rs201365646	ENSP00000230480	R108G	T	0.24	3	B	0.001	3	B	0.001	3
rs201786558	ENSP00000317598	A28G/V	D	0	1	Prob. D	0.998	1	Prob. D	0.992	1
rs202125661	ENSP00000421561	S366Y	D	0	1	B	0.001	3	B	0.001	3
rs267601048	ENSP00000361125	S338F	T	0.69	3	Prob. D	0.998	1	Prob. D	0.846	2
rs367757959	ENSP00000230480	R80Q	D	0.05	1	B	0.35	3	B	0.284	3
rs368064502	ENSP00000317598	S77N	T	0.13	3	Poss. D	0.578	2	B	0.264	3
rs368256497	ENSP00000230480	G56R	D	0	1	Poss. D	0.955	1	Poss. D	0.877	2
rs368814156	ENSP00000230480	N60D	T	0.21	3	Poss. D	0.482	2	Poss. D	0.597	2
rs369593555	ENSP00000230480	R143L	D	0.01	1	Poss. D	0.916	2	Poss. D	0.458	2
rs371177206	ENSP00000361125	R335C	T	0.29	3	Poss. D	0.868	2	B	0.142	3
rs371208770	ENSP00000230480	K160R	D	0.03	1	B	0.159	3	B	0.111	3
rs372731987	ENSP00000317598	R165Q	T	0.08	2	Prob. D	0.994	1	Poss. D	0.49	2
rs373521056	ENSP00000230480	R110S	T	0.25	3	B	0.006	3	B	0.007	3
rs374057152	ENSP00000230480	M53T	T	0.24	3	B	0.001	3	B	0.01	3
rs374253522	ENSP00000230480	R163Q	D	0	1	Prob. D	1	1	Prob. D	0.999	1
rs374420337	ENSP00000230480	C58Y	D	0	1	Prob. D	1	1	Prob. D	1	1
rs374451231	ENSP00000317598	R176P	D	0	1	Prob. D	0.999	1	Prob. D	0.969	1
rs375450405	ENSP00000317598	Y197C	T	0.1	2	B	0.108	3	B	0.026	3
rs376388064	ENSP00000230480	P130L	T	0.1	2	B	0.316	3	B	0.035	3
rs541717889	ENSP00000317598	C61S	T	0.39	3	B	0.019	3	B	0.01	3
rs554561071	ENSP00000230480	I27V	T	0.69	3	B	0.001	3	B	0.021	3
rs555315943	ENSP00000230480	H10Q	T	0.43	3	B	0.19	3	B	0.051	3

rs569791806	ENSP00000230480	G6R	T	0.28	3	B	0.147	3	B	0.252	3
rs574579489	ENSP00000317598	V45G	D	0.01	1	B	0.004	3	B	0.007	3
rs745387465	ENSP00000317598	A150S	T	0.13	3	B	0.094	3	B	0.019	3
rs746680821	ENSP00000317598	R141Q	D	0.01	1	Prob. D	0.995	1	Poss. D	0.714	2
rs747915464	ENSP00000317598	S11N	D	0	1	B	0.13	3	B	0.052	3
rs747966855	ENSP00000317598	A47V	T	0.21	3	Poss. D	0.906	2	Poss. D	0.501	2
rs748352475	ENSP00000230480	P38A	T	0.11	3	Prob. D	0.995	1	Prob. D	0.98	1
rs748710411	ENSP00000317598	P174L	D	0	1	Poss. D	0.557	2	B	0.206	3
rs748846771	ENSP00000317598	Y197N	T	0.08	2	Poss. D	0.731	2	B	0.242	3
rs748984440	ENSP00000230480	D159A	D	0.04	1	Prob. D	0.982	1	Poss. D	0.792	2
rs749282093	ENSP00000317598	P34S	D	0	1	Poss. D	0.827	2	B	0.342	3
rs749491856	ENSP00000230480	N152H	D	0	1	Prob. D	0.998	1	Prob. D	0.918	2
rs750127977	ENSP00000361125	K337I	T	0.29	3	Prob. D	0.996	1	Poss. D	0.899	2
rs750662808	ENSP00000317598	S85A	D	0	1	Poss. D	0.514	2	B	0.261	3
rs751392131	ENSP00000317598	S168N	D	0.03	1	Poss. D	0.557	2	B	0.172	3
rs751447901	ENSP00000317598	F183L	T	0.15	3	B	0.036	3	B	0.041	3
rs751944345	ENSP00000317598	R22Q	D	0.05	1	B	0	3	B	0	3
rs752674861	ENSP00000317598	T120M	D	0	1	Prob. D	1	1	Prob. D	0.998	1
rs752729944	ENSP00000317598	P163R	D	0.01	1	B	0.004	3	B	0.009	3
rs752907384	ENSP00000230480	R154C	D	0.01	1	Prob. D	1	1	Prob. D	0.985	1
rs753997360	ENSP00000317598	R110L	D	0.01	1	Poss. D	0.766	2	Poss. D	0.496	2
rs754375185	ENSP00000317598	H199Y	T	0.18	3	B	0.0239	3	B	0.098	3
rs755000167	ENSP00000317598	P36S	T	0.22	3	B	0.137	3	B	0.075	3
rs755046960	ENSP00000317598	G112R	D	0	1	Prob. D	1	1	Prob. D	1	1
rs755307045	ENSP00000230480	V31M	D	0	1	Prob. D	1	1	Prob. D	0.993	1
rs755361036	ENSP00000230480	T69S	D	0.02	1	B	0.012	3	B	0.052	3
rs755797583	ENSP00000317598	L196V/F	D	0.02	1	Poss. D	0.729	2	Poss. D	0.639	2
rs756152837	ENSP00000317598	D3N	D	0	1	Prob. D	0.996	1	Prob. D	0.99	1
rs756193427	ENSP00000230480	E91A	D	0.02	1	B	0.361	3	B	0.309	3
rs756210326	ENSP00000317598	R30G	T	0.11	3	B	0.003	3	B	0.004	3
rs756935833	ENSP00000317598	A169V	D	0.01	1	Prob. D	0.994	1	Prob. D	0.736	2
rs757103787	ENSP00000317598	W187R	D	0	1	Poss. D	0.732	2	Poss. D	0.556	2
rs758505735	ENSP00000230480	P114H	T	0.31	3	B	0	3	B	0.001	3
rs758648305	ENSP00000230480	I74T	D	0.02	1	B	0.003	3	B	0.026	3
rs759253179	ENSP00000230480	V50M	D	0	1	Prob. D	0.999	1	Prob. D	0.993	1
rs759806982	ENSP00000317598	S178A	T	0.26	3	B	0.015	3	B	0.011	3
rs759826070	ENSP00000230480	E153Q	T	0.06	2	Prob. D	0.999	1	Prob. D	0.978	1

rs760103506	ENSP00000317598	P18R	D	0	1	Poss. D	0.703	2	B	0.24	3
rs760891966	ENSP00000230480	E112V	D	0	1	B	0.446	3	B	0.197	3
rs760903464	ENSP00000317598	P160R/L	D	0.01	1	B	0.358	3	B	0.221	3
rs761101895	ENSP00000317598	G175C	D	0.02	1	Prob. D	1	1	Prob. D	0.999	1
rs761835228	ENSP00000230480	H10Y	T	1	3	B	0.001	3	B	0.002	3
rs762362569	ENSP00000317598	E156D	D	0.05	1	B	0.318	3	B	0.109	3
rs762664023	ENSP00000230480	R54Q	D	0	1	Prob. D	1	1	Prob. D	0.997	1
rs763031344	ENSP00000361125	K334E	T	0.15	3	Prob. D	0.983	1	Prob. D	0.798	2
rs764480708	ENSP00000317598	R22W	D	0.02	1	B	0.148	3	B	0.005	3
rs765392517	ENSP00000317598	S162N	D	0.02	1	Poss. D	0.531	2	B	0.145	3
rs765514291	ENSP00000230480	E153D	T	0.13	3	Prob. D	0.997	1	Prob. D	0.936	2
rs766474822	ENSP00000317598	G95E	T	0.14	3	B	0.003	3	B	0.006	3
rs766541343	ENSP00000230480	N113S	T	0.84	3	B	0.009	3	B	0.027	3
rs767279692	ENSP00000317598	H189R	D	0.04	1	B	0.022	3	B	0.026	3
rs767587788	ENSP00000230480	I81L	T	0.16	3	B	0.001	3	B	0.013	3
rs767638850	ENSP00000317598	A26P/S	D	0	1	Prob. D	1	1	Prob. D	0.998	1
rs767672556	ENSP00000421561	R369G	D	0.01	1	B	0	3	B	0	3
rs767781830	ENSP00000230480	A109T	T	0.58	3	B	0	3	B	0.005	3
rs768366559	ENSP00000230480	P161L	T	0.68	3	Prob. D	0.999	1	Prob. D	0.935	2
rs768628753	ENSP00000317598	R105S	D	0.03	1	B	0.4	3	B	0.167	3
rs769291960	ENSP00000317598	R152Q	T	0.61	3	B	0.001	3	B	0.001	3
rs769584601	ENSP00000230480	K138R	T	0.36	3	B	0.022	3	B	0.049	3
rs769625979	ENSP00000230480	P51S	T	0.73	3	B	0.003	3	B	0.015	3
rs770279324	ENSP00000230480	E3G	T	0.28	3	B	0	3	B	0	3
rs770795261	ENSP00000230480	I44M	T	0.19	3	Poss. D	0.932	2	Poss. D	0.889	2
rs771561387	ENSP00000230480	R163W	D	0	1	Prob. D	1	1	Prob. D	1	1
rs771641958	ENSP00000317598	S11R	T	0.07	2	B	0	3	B	0.001	3
rs771830463	ENSP00000317598	F55S	D	0	1	Prob. D	0.999	1	Prob. D	0.996	1
rs772184987	ENSP00000230480	P38L	D	0.01	1	Prob. D	1	1	Prob. D	0.999	1
rs772365349	ENSP00000317598	S157G	D	0.02	1	B	0.009	3	B	0.013	3
rs772861763	ENSP00000317598	H15Y	D	0	1	Prob. D	0.989	1	Prob. D	0.969	1
rs774260380	ENSP00000317598	P10S	D	0	1	Prob. D	1	1	Prob. D	0.996	1
rs774265827	ENSP00000361125	R335H	T	0.16	3	Prob. D	0.999	1	Prob. D	0.934	2
rs774898653	ENSP00000317598	E155K	D	0.02	1	B	0.394	3	B	0.042	3
rs775464274	ENSP00000361125	R333Q	T	0.19	3	Prob. D	0.993	1	Poss. D	0.735	2
rs775918375	ENSP00000317598	R20Q	T	0.13	3	Prob. D	0.994	1	Poss. D	0.566	2
rs777632185	ENSP00000230480	R121W	D	0	1	Prob. D	1	1	Prob. D	0.991	1

rs776522854	ENSP00000230480	T132M	D	0.01	1	Prob. D	1	1	Prob. D	0.996	1
rs776087137	ENSP00000317598	E75K	T	0.79	3	B	0.025	3	B	0.006	3
rs779923978	ENSP00000317598	D7N/H	D	0	1	Prob. D	0.996	1	Prob. D	0.99	1
rs777797068	ENSP00000230480	I41F	D	0	1	B	0.363	3	Poss. D	0.555	2
rs778942294	ENSP00000317598	V45I	D	0.01	1	Poss. D	0.872	2	B	0.392	3
rs914956206	ENSP00000317598	G19V	D	0	1	Prob. D	1	1	Prob. D	0.999	1
rs780163839	ENSP00000317598	R30H	D	0	1	Prob. D	0.99	1	Poss. D	0.616	2
rs780766978	ENSP00000317598	R148K	T	0.19	3	B	0.039	3	B	0.035	3
rs866329410	ENSP00000230480	A146V	T	0.12	3	Poss. D	0.789	2	B	0.109	3
rs897547135	ENSP00000317598	A154V	D	0	1	B	0.386	3	B	0.059	3
rs910906787	ENSP00000230480	I44F	D	0.03	1	Poss. D	0.932	2	Poss. D	0.733	2
rs942729487	ENSP00000317598	S13N	D	0	1	Prob. D	0.989	1	Prob. D	0.969	1
rs915247074	ENSP00000317598	A113P	D	0	1	Poss. D	0.739	2	Poss. D	0.505	2
rs916589417	ENSP00000317598	G44R	D	0	1	Prob. D	0.989	1	Prob. D	0.911	2
rs921621484	ENSP00000361125	S323P	T	0.2	3	B	0.148	3	B	0.134	3
rs922704212	ENSP00000317598	E104G	D	0	1	Poss. D	0.59	2	B	0.223	3
rs926455673	ENSP00000317598	A132G	T	0.11	3	Poss. D	0.827	2	B	0.423	3
rs928130394	ENSP00000317598	A172V	D	0.04	1	Poss. D	0.6	2	B	0.215	3
rs930605708	ENSP00000317598	G44E	T	0.21	3	Prob. D	0.989	1	Poss. D	0.884	2
rs933893718	ENSP00000230480	E42K	D	0.01	1	Poss. D	0.917	2	Poss. D	0.657	2
rs939211010	ENSP00000317598	A133S	D	0.01	1	B	0.419	3	B	0.3	3
rs946601059	ENSP00000317598	R114P	D	0.01	1	B	0.426	3	B	0.239	3
rs964195968	ENSP00000317598	L192R	D	0	1	B	0.034	3	B	0.07	3
rs964280210	ENSP00000317598	V42G	D	0	1	Poss. D	0.904	2	B	0.402	3
rs1004481569	ENSP00000317598	G72W	D	0	1	Prob. D	0.998	1	Prob. D	0.989	1
rs985359727	ENSP00000230480	Y43F	T	0.12	3	B	0	3	B	0.003	3
rs988521895	ENSP00000317598	G106R	D	0	1	B	0.006	3	B	0.009	3
rs1003930162	ENSP00000230480	S119L	D	0.04	1	Prob. D	0.982	1	Poss. D	0.792	2
rs1027989270	ENSP00000317598	T8I	D	0	1	Prob. D	0.999	1	Prob. D	0.991	1
rs1005693041	ENSP00000230480	V18I	D	0.04	1	Poss. D	0.899	2	Poss. D	0.723	2
rs1029847964	ENSP00000317598	H200L	D	0.03	1	B	0.05	3	B	0.015	3
rs1044836001	ENSP00000317598	P38R	D	0	1	Prob. D	0.971	1	Poss. D	0.775	2
rs1179946537	ENSP00000317598	E97K	D	0	1	Prob. D	0.996	1	Prob. D	0.99	1
rs1047373716	ENSP00000230480	V13M	D	0.04	1	B	0.011	3	B	0.024	3
rs1049045355	ENSP00000317598	T180A	T	0.07	2	B	0.341	3	B	0.082	3
rs1049631147	ENSP00000317598	G46R	D	0	1	Prob. D	0.998	1	Poss. D	0.854	2
rs1164380167	ENSP00000317598	G117A	T	0.13	3	Prob. D	0.97	1	Poss. D	0.679	2

rs1166395511	ENSP00000230480	R103K	D	0.01	1	Poss. D	0.795	2	Poss. D	0.615	2
rs1168841710	ENSP00000317598	R48G	D	0	1	B	0.009	3	B	0.011	3
rs1169884447	ENSP00000317598	P38S	D	0	1	Poss. D	0.827	2	B	0.342	3
rs1170711953	ENSP00000317598	Q32H	D	0	1	Poss. D	0.95	1	Poss. D	0.648	2
rs1170737707	ENSP00000230480	R108T	T	0.49	3	B	0.001	3	B	0.002	3
rs1176966214	ENSP00000230480	H124R	T	0.9	3	B	0.004	3	B	0.006	3
rs1179956540	ENSP00000317598	A113D	D	0	1	B	0.241	3	B	0.109	3
rs1187507161	ENSP00000230480	T69A	D	0.05	1	B	0.028	3	B	0.12	3
rs1323880331	ENSP00000317598	A130V	D	0	1	Poss. D	0.93	2	Poss. D	0.548	2
rs1181812356	ENSP00000317598	A28T	D	0	1	Prob. D	0.996	1	Prob. D	0.99	1
rs1325785128	ENSP00000317598	F55L	D	0	1	Prob. D	0.996	1	Prob. D	0.98	1
rs1324376559	ENSP00000317598	R63C	D	0	1	Prob. D	0.999	1	Poss. D	0.882	2
rs1328421749	ENSP00000317598	L139V	T	0.12	3	B	0.122	3	B	0.088	3
rs1329473200	ENSP00000230480	Q96R	D	0	1	Poss. D	0.875	2	Poss. D	0.772	2
rs1331499170	ENSP00000317598	A135S	D	0.01	1	Prob. D	0.971	1	Poss. D	0.532	2
rs1188254133	ENSP00000230480	L125V	T	0.08	2	Prob. D	0.985	1	Poss. D	0.871	2
rs1189544809	ENSP00000230480	R147M	D	0	1	Prob. D	0.999	1	Prob. D	0.95	1
rs1193943585	ENSP00000230480	E11K	T	0.48	3	B	0.026	3	B	0.01	3
rs1193726870	ENSP00000317598	G112D/V	D	0	1	Prob. D	1	1	Prob. D	0.999	1
rs1194395358	ENSP00000317598	G65R	D	0.01	1	Prob. D	0.989	1	Poss. D	0.8	2
rs1201834176	ENSP00000317598	P208T	D	0.04	1	B	0.035	3	B	0.066	3
rs1201894677	ENSP00000317598	E43K	D	0	1	Poss. D	0.906	2	B	0.418	3
rs1202242670	ENSP00000317598	R63L	T	0.18	3	B	0.02	3	B	0.02	3
rs1203752241	ENSP00000317598	A154P	D	0.01	1	B	0.01	3	B	0.005	3
rs1208889729	ENSP00000230480	R21C	D	0	1	Prob. D	0.998	1	Poss. D	0.886	2
rs1212415280	ENSP00000317598	A142S	T	0.06	2	Poss. D	0.93	2	B	0.38	3
rs1217657176	ENSP00000317598	G147R	D	0.01	1	B	0.005	3	B	0.005	3
rs1218363085	ENSP00000317598	G31R	D	0	1	Prob. D	0.993	1	Poss. D	0.672	2
rs1219395046	ENSP00000317598	P90S	D	0	1	Prob. D	0.995	1	Poss. D	0.891	2
rs1219959057	ENSP00000317598	G173R	D	0.01	1	Prob. D	0.989	1	Poss. D	0.868	2
rs1221931208	ENSP00000317598	C61R	D	0	1	Poss. D	0.835	2	Poss. D	0.499	2
rs1222213359	ENSP00000317598	R87Q	T	0.2	3	Poss. D	0.655	2	B	0.093	3
rs1229919945	ENSP00000230480	S142P	T	0.27	3	Poss. D	0.904	2	Poss. D	0.514	2
rs1230026567	ENSP00000317598	S164R	D	0	1	Poss. D	0.681	2	B	0.192	3
rs1236934940	ENSP00000317598	A169P	D	0.01	1	Prob. D	0.999	1	Prob. D	0.943	2
rs1236943525	ENSP00000317598	R165G	D	0	1	Poss. D	0.866	2	B	0.345	3
rs1240763998	ENSP00000317598	P136A	D	0	1	B	0.04	3	B	0.03	3

rs1243046808	ENSP00000317598	P18A	T	0.56	3	B	0.001	3	B	0.003	3
rs1245747754	ENSP00000317598	R110G	D	0.03	1	Poss. D	0.766	2	B	0.414	3
rs1248736644	ENSP00000317598	R4G	D	0	1	Prob. D	0.989	1	Prob. D	0.978	1
rs1255510121	ENSP00000230480	C135Y	T	0.16	3	Prob. D	1	1	Prob. D	0.999	1
rs1258102197	ENSP00000317598	A140T	D	0	1	Prob. D	0.989	1	Poss. D	0.732	2
rs1260801025	ENSP00000317598	R151H	D	0.01	1	Prob. D	0.957	1	B	0.385	3
rs1261671668	ENSP00000230480	I74V	T	1	3	B	0	3	B	0.002	3
rs1267535717	ENSP00000230480	R21H	D	0.02	1	Poss. D	0.801	2	B	0.199	3
rs1269130764	ENSP00000421561	L367P	D	0.01	1	Prob. D	0.993	1	Poss. D	0.884	2
rs1271681344	ENSP00000317598	L59R	D	0	1	Prob. D	0.999	1	Prob. D	0.999	1
rs1272402969	ENSP00000230480	P161A	T	0.09	2	Prob. D	0.996	1	Poss. D	0.899	2
rs1274049043	ENSP00000317598	G39R	T	0.24	3	Prob. D	0.987	1	Poss. D	0.555	2
rs1274302722	ENSP00000230480	S142L	T	1	3	B	0.01	3	B	0.002	3
rs1275152500	ENSP00000230480	G86S	T	1	3	B	0	3	B	0.001	3
rs1277055983	ENSP00000361125	Q331H	T	0.4	3	Prob. D	0.998	1	Prob. D	0.93	2
rs1277821911	ENSP00000317598	A80T	D	0.01	1	B	0.296	3	B	0.039	3
rs1278594233	ENSP00000230480	M1T	D	0	1	B	0	3	B	0	3
rs1282837263	ENSP00000317598	F64S	T	0.57	3	B	0	3	B	0	3
rs1284410244	ENSP00000230480	Q85R	T	0.64	3	B	0	3	B	0.005	3
rs1287008224	ENSP00000317598	R171L	T	0.33	3	B	0	3	B	0.001	3
rs1287276985	ENSP00000230480	C135S	T	0.45	3	Prob. D	1	1	Prob. D	0.999	1
rs1295232450	ENSP00000230480	N139S	T	0.4	3	B	0.215	3	B	0.101	3
rs1295263390	ENSP00000317598	V149L	T	0.71	3	B	0.007	3	B	0.013	3
rs1306796730	ENSP00000317598	T6A	D	0	1	Prob. D	0.99	1	Prob. D	0.971	1
rs1313986907	ENSP00000317598	S129R	D	0	1	Prob. D	0.999	1	Prob. D	0.996	1
rs1317828597	ENSP00000317598	A9T	D	0	1	Prob. D	0.999	1	Prob. D	0.99	1
rs1302730981	ENSP00000317598	S170N	D	0.01	1	Poss. D	0.557	2	B	0.157	3
rs1320240999	ENSP00000317598	G41A	D	0	1	Poss. D	0.869	2	Poss. D	0.451	2
rs1333329217	ENSP00000317598	A123T	D	0	1	Poss. D	0.554	2	B	0.197	3
rs1333365032	ENSP00000230480	Q111R	T	0.1	2	B	0.075	3	B	0.201	3
rs1334033917	ENSP00000230480	E70K	T	0.24	3	B	0	3	B	0.001	3
rs1335264265	ENSP00000317598	P107T	D	0.01	1	Prob. D	1	1	Prob. D	0.998	1
rs1339630870	ENSP00000317598	R134C	D	0	1	B	0.16	3	B	0.037	3
rs1339912264	ENSP00000317598	P90R	D	0	1	Prob. D	0.998	1	Poss. D	0.742	2
rs1341479209	ENSP00000317598	V149G	T	0.79	3	B	0.337	3	B	0.227	3
rs1342583213	ENSP00000230480	Q77E	T	0.39	3	B	0	3	B	0.001	3
rs1344464381	ENSP00000317598	S168R	D	0.02	1	B	0	3	B	0	3

rs1346131223	ENSP00000230480	T75A	T	0.08	2	B	0	3	B	0.008	3
rs1348253363	ENSP00000317598	L139P	T	0.21	3	Poss. D	0.615	2	B	0.214	3
rs1349996970	ENSP00000317598	A83V	T	0.1	2	B	0.002	3	B	0.004	3
rs1351424000	ENSP00000317598	E93D	D	0.01	1	B	0.135	3	B	0.101	3
rs1355739548	ENSP00000317598	A193T	D	0.01	1	B	0.135	3	B	0.101	3
rs1355913467	ENSP00000317598	G33V	D	0.04	1	Prob. D	0.964	1	Poss. D	0.885	2
rs1357683782	ENSP00000230480	F15I	D	0	1	B	0.411	3	Poss. D	0.497	2
rs1357987443	ENSP00000230480	M53L	T	0.34	3	Poss. D	0.672	2	B	0.273	3
rs1359856307	ENSP00000230480	K46Q	T	0.27	3	B	0	3	B	0.001	3
rs1364620045	ENSP00000317598	E179D	D	0.01	1	B	0.062	3	B	0.031	3
rs1365501228	ENSP00000317598	A9V	D	0	1	Poss. D	0.718	2	B	0.157	3
rs1370001698	ENSP00000317598	P116L	D	0	1	Prob. D	0.996	1	Prob. D	0.986	1
rs1372773191	ENSP00000317598	A142D	D	0	1	Prob. D	1	1	Prob. D	0.998	1
rs1381796343	ENSP00000317598	G146E	T	0.43	3	Poss. D	0.93	2	Poss. D	0.623	2
rs1381953838	ENSP00000317598	G146R	D	0.01	1	B	0.004	3	B	0.003	3
rs1383496488	ENSP00000317598	P34L	D	0	1	B	0.004	3	B	0.005	3
rs138421	ENSP00000317598	E73K	T	0.11	3	Prob. D	0.989	1	Poss. D	0.564	2
rs1388776124	ENSP00000317598	E100D	D	0	1	B	0.016	3	B	0.015	3
rs1391776661	ENSP00000317598	E100G	D	0	1	Poss. D	0.514	2	B	0.261	3
rs1395807781	ENSP00000361125	R325P	T	0.34	3	Prob. D	0.993	1	Poss. D	0.795	2
rs1397698749	ENSP00000361125	K329R	T	0.22	3	Poss. D	0.931	2	Poss. D	0.628	2
rs1404013760	ENSP00000317598	G144A	T	1	3	Poss. D	0.72	2	B	0.429	3
rs1404494791	ENSP00000317598	G40A	D	0	1	B	0	3	B	0	3
rs1406201670	ENSP00000317598	A51S	D	0	1	Prob. D	0.999	1	Prob. D	0.996	1
rs1412676860	ENSP00000230480	N98D	D	0.03	1	Poss. D	0.537	2	Poss. D	0.489	2
rs1419531239	ENSP00000317598	T180I	D	0.01	1	Poss. D	0.924	2	Poss. D	0.495	2
rs1421145908	ENSP00000230480	E62K	D	0	1	B	0.14	3	B	0.063	3
RS1422237480	ENSP00000317598	E103K	D	0	1	Poss. D	0.557	2	B	0.157	3
rs1423832768	ENSP00000317598	E96D	T	0.15	3	Poss. D	0.59	2	B	0.308	3
rs1426591848	ENSP00000317598	A140G	D	0	1	B	0.373	3	B	0.205	3
rs1426761489	ENSP00000317598	G49R	D	0	1	Prob. D	0.964	1	Poss. D	0.696	2
rs1429874344	ENSP00000230480	R121Q	T	0.06	2	Prob. D	0.999	1	Prob. D	0.987	1
rs1434509876	ENSP00000317598	E89K	T	0.51	3	Prob. D	0.992	1	Prob. D	0.918	2
rs1443465532	ENSP00000230480	A2P	T	1	3	B	0.045	3	B	0.031	3
rs1443899148	ENSP00000317598	R114W	T	0.08	2	B	0	3	B	0.001	3
rs1447433103	ENSP00000317598	G153S	T	0.18	3	Poss. D	0.95	1	B	0.427	3
rs1452295912	ENSP00000317598	S164T	D	0.05	1	Poss. D	0.573	2	B	0.202	3

rs1456457746	ENSP00000230480	G90R	T	0.35	3	B	0	3	B	0	3
rs1457663984	ENSP00000317598	Q137R	D	0.03	1	B	0.002	3	B	0.006	3
rs1459669662	ENSP00000230480	E65G	D	0	1	B	0.013	3	B	0.026	3
rs1465046427	ENSP00000230480	K123M	D	0	1	B	0.385	3	B	0.366	3
rs1466363160	ENSP00000317598	S143T	T	0.07	2	Poss. D	0.844	2	B	0.431	3
rs1469291769	ENSP00000317598	R145G	T	0.09	2	B	0.218	3	B	0.059	3
rs1470144810	ENSP00000317598	E94D	D	0.03	1	B	0.017	3	B	0.025	3
rs1475211079	ENSP00000361125	K332T	T	0.31	3	B	0.175	3	B	0.135	3
rs1475279373	ENSP00000230480	G116R	T	0.47	3	Prob. D	1	1	Prob. D	0.971	1
rs1478764872	ENSP00000317598	G72E	D	0	1	B	0.275	3	B	0.076	3
rs1490238295	ENSP00000317598	P174S	T	0.23	3	Prob. D	0.961	1	Poss. D	0.804	2

*T=Tolerate, D=Deleterious, B=Benign, Prob.D= Probably Damaging, Poss. D= Possibly Damaging

*Here categories SNPs into Class:

1. SIFT database: SIFT Score is 0.0: Class 1
   SIFT Score is 0.01- 0.05: Class 2
   SIFT score is 0.06-1.00: Class 3
2. PolyPhen 2 database:
   HumDiv and HumVar score is 0.9-1.0: Class 1
   HumDiv and HumVar score is 0.15-0.85: Class 2
   HumDiv and HumVar score is 0.00 - 0.15: Class 3

Table S3: List of SNPs predicted by PredictSNP, MAPP, PhD-SNP, PolyPhen-1, SNAP, nsSNP-Analyzer, PANTHER and SNPs&GO. (N = Neutral, D = Deleterious, U = Unknown, N/A= Not available)

**Protein ID**	**Mutation**	**PredictSNP**	**MAPP**	**PhD-SNP**	**PolyPhen-1**	**SNAP**	**nsSNP-Analyzer**	**PANTHER**	**SNPs&GO**
ENSP00000230480	R80W	D	D	D	D	D	D	D	Neutral
**ENSP00000230480**	**R54Q**	**D**	**D**	**D**	**D**	**D**	**D**	**D**	**Disease**
**ENSP00000230480**	**C58Y**	**D**	**D**	**D**	**D**	**D**	**D**	**D**	**Disease**
**ENSP00000230480**	**V31M**	**D**	**D**	**D**	**D**	**D**	**D**	**D**	**Disease**
**ENSP00000230480**	**V50M**	**D**	**D**	**D**	**D**	**D**	**D**	**D**	**Disease**
**ENSP00000230480**	**P38L**	**D**	**D**	**D**	**D**	**D**	**D**	**D**	**Disease**
ENSP00000230480	R121W	D	N/A	D	D	D	N	D	Neutral
ENSP00000230480	R147M	D	D	N	D	D	D	U	Neutral

ENSP00000361 125	R339W	D	N/A	D	D	D	U	U	Neutral
ENSP00000230 480	R154C	D	N/A	D	D	D	N	U	Neutral
ENSP00000230 480	R163W	D	D	N	D	D	N	U	Neutral
ENSP00000317 598	F55S	D	N/A	D	D	D	U	U	Neutral
ENSP00000317 598	L59R	D	N/A	D	D	D	U	U	Neutral
ENSP00000317 598	A28G/V	D	N/A	N	N/D	D	U	U	Neutral
ENSP00000230 480	R163Q	D	N	N	D	D	N	U	Neutral
ENSP00000317 598	R176P	D	N/A	N	D	D	U	U	Neutral
ENSP00000317 598	T120M	D	N/A	N	D	D	U	U	Neutral
ENSP00000317 598	G112R	D	N/A	N	D	D	U	U	Neutral
ENSP00000317 598	D3N	D	N/A	N	D	D	U	U	Neutral
ENSP00000317 598	H15Y	D	N/A	N	D	D	U	U	Neutral
ENSP00000230 480	T132M	D	N/A	N	D	D	N	U	Neutral
ENSP00000317 598	D7N/H	D	N/A	N	D	D	U	U	Neutral
ENSP00000317 598	G19V	D	N/A	N	D	D	U	U	Neutral
ENSP00000317 598	S13N	D	N/A	N	D	D	U	U	Neutral
ENSP00000317 598	G72W	D	N/A	N	D	D	U	U	Neutral
ENSP00000317 598	T8I	D	N/A	N	D	D	U	U	Neutral
ENSP00000317 598	E97K	D	N/A	N	D	D	U	U	Neutral
ENSP00000317 598	A28T	D	N/A	N	D	D	U	U	Neutral
ENSP00000317 598	G112D/V	D	N/A	N	D	D	U	U	Neutral
ENSP00000317 598	R4G	D	N/A	N	D	D	U	U	Neutral
ENSP00000317 598	S129R	D	N/A	N	D	D	U	U	Neutral

ENSP00000317598	P116L	D	N/A	N	D	D	U	U	Neutral
ENSP00000317598	A142D	D	N/A	N	D	D	U	U	Neutral
ENSP00000317598	A51S	D	N/A	N	D	D	U	U	Neutral
ENSP00000317598	G175C	D	N/A	N	D	N	U	U	Neutral
ENSP00000317598	A26P/S	D/N	N/A	N	D/N	N	U	U	Neutral
ENSP00000317598	P10S	D	N/A	N	D	N	U	U	Neutral
ENSP00000317598	T6A	D	N/A	N	N	D	U	U	Neutral
ENSP00000317598	A9T	D	N/A	N	D	N	U	U	Neutral
ENSP00000317598	P107T	D	N/A	N	D	N	U	U	Neutral
ENSP00000230480	R154H	N	N/A	N	N	N	N	U	Neutral
ENSP00000317598	F55L	N	N/A	N	N	N	U	U	Neutral

Table S4: Protein stability changes due to mutations predicted by I-Mutant Suite; DDG<0: Decrease Stability; DDG>0: Increase Stability; RI: Reliability Index.)

rsID	Protein ID	Mutation	Sign DDG	DDG value prediction(kcal/mol)	RI
rs374420337	ENSP00000230480	C266Y	Decrease	-0.19	1
rs772184987	ENSP00000230480	P246L	Decrease	-0.21	2
rs762664023	ENSP00000230480	R262Q	Decrease	-0.76	8
rs759253179	ENSP00000230480	V258M	Decrease	-0.68	7
rs755307045	ENSP00000230480	V239M	Decrease	-0.73	6

Table S5: Dimer Interface H-bond and Salt bridge Interactions.

	Hydrogen Bond			Salt Bridge		
	A-Chain	B-Chain	Distance(Å)	A-Chain	B-Chain	Distance(Å)
**Native**	A:Thr283[O]<br>A:Gln285[OE1]<br>A:Glu236[OE1]<br>A:Cys257[SG]<br>A:Ser256[OG]<br>A:Val221[O]<br>A:Gln285[N]<br>A:Ser256[N]<br>A:Cys257[N]<br>A:Val221[N]	B:Val221[N]<br>B:Phe223[N]<br>B:Arg229[HH21]<br>B:Cys266[SG]<br>B:Cys257[SG]<br>B:Gln285[N]<br>B:Val221[O]<br>B:Cys266[SG]<br>B:Cys266[SG]<br>B:Thr283[O]	2.94<br>3.45<br>2.20<br>3.39<br>3.55<br>2.91<br>2.78<br>2.75<br>3.65<br>2.87	A:Glu236[OE1]	B:Arg229[NH2]	3.13
**R262Q**	A:Thr283[O]<br>A:Glu236[OE2]<br>A:Glu236[OE1]<br>A:Cys266[SG]<br>A:Cys266[SG]<br>A:Cys266[SG]<br>A:Cys266[SG]<br>A:Cys257[O]<br>A:Val221[O]<br>A:Val221[N]<br>A:Phe223[N]<br>A:Ser230[OG]<br>A:Ser256[N]<br>A:Cys266[SG]<br>A:Gln285[N]	B:Val221[N]<br>B:Arg229[HH12]<br>B:Arg229[HH22]<br>B:Ser256[N]<br>B:Ser256[SG]<br>B:Cys257[N]<br>B:Cys257[SG]<br>B:Cys266[SG]<br>B:Gln285[N]<br>B:Thr283[O]<br>B:Gln285[OE1]<br>B:Cys257[O]<br>B:Cys266[SG]<br>B:Cys257[O]<br>B:Val221[O]	3.07<br>1.99<br>1.65<br>3.58<br>3.70<br>3.44<br>3.78<br>3.71<br>2.81<br>3.05<br>3.21<br>2.80<br>3.82<br>3.76<br>2.88	A:Glu236[OE1]<br>A:Glu236[OE2]<br>A:Glu236[OE1]<br>A:Glu236[OE2]	B:Arg229[NH1]<br>B:Arg229[NH1]<br>B:Arg229[NH2]<br>B:Arg229[NH2]	3.38<br>2.98<br>2.63<br>3.70
**C266Y**	A:Thr283[O]<br>A:Val221[O]<br>A:Val221[N]<br>A:Ser230[OG]<br>A:Lys254[HZ3]<br>A:Lys254[HZ2]<br>A:Gln285[N]	B:Val221[N]<br>B:Gln285[N]<br>B:Thr283[O]<br>B:Cys257[O]<br>B:Tyr266[OH]<br>B:Asn268[OD1]<br>B:Val221[O]	2.95<br>2.64<br>2.90<br>2.57<br>2.44<br>1.76<br>2.96			
